# Repeated Sessions of Transcranial Direct Current Stimulation on Adolescents With Autism Spectrum Disorder: Study Protocol for a Randomized, Double-Blind, and Sham-Controlled Clinical Trial

**DOI:** 10.3389/fpsyt.2021.680525

**Published:** 2021-08-30

**Authors:** Karin Prillinger, Stefan T. Radev, Gabriel Amador de Lara, Manfred Klöbl, Rupert Lanzenberger, Paul L. Plener, Luise Poustka, Lilian Konicar

**Affiliations:** ^1^Department of Child and Adolescent Psychiatry, Medical University of Vienna, Vienna, Austria; ^2^Institute of Psychology, University of Heidelberg, Heidelberg, Germany; ^3^Department of Psychiatry and Psychotherapy, Medical University of Vienna, Vienna, Austria; ^4^Department of Child and Adolescent Psychiatry and Psychotherapy, University of Ulm, Ulm, Germany; ^5^Department of Child and Adolescent Psychiatry and Psychotherapy, University Medical Center Göttingen, Göttingen, Germany

**Keywords:** autism spectrum disorder, transcranial DC stimulation, clinical trial, social cognition, neuromodulation

## Abstract

**Background:** Social–emotional difficulties are a core symptom of autism spectrum disorder (ASD). Accordingly, individuals with ASD have problems with social cognition such as recognizing emotions from other peoples' faces. Various results from functional magnetic resonance imaging and electroencephalography studies as well as eye-tracking data reveal a neurophysiological basis of these deficits by linking them to abnormal brain activity. Thus, an intervention targeting the neural origin of ASD impairments seems warranted. A safe method able to influence neural activity is transcranial direct current stimulation (tDCS). This non-invasive brain stimulation method has already demonstrated promising results in several neuropsychiatric disorders in adults and children. The aim of this project is to investigate the effects of tDCS on ASD symptoms and their neural correlates in children and adolescents with ASD.

**Method:** This study is designed as a double-blind, randomized, and sham-controlled trial with a target sample size of 20 male participants (aged 12–17 years) diagnosed with ASD. Before randomization, the participants will be stratified into comorbid depression, comorbid ADHS/conduct disorder, or no-comorbidity groups. The intervention phase comprises 10 sessions of anodal or sham tDCS applied over the left prefrontal cortex within 2 consecutive weeks. To engage the targeted brain regions, participants will perform a social cognition training during the stimulation. TDCS-induced effects on ASD symptoms and involved neural circuits will be investigated through psychological, neurophysiological, imaging, and behavioral data at pre- and post-measurements. Tolerability will be evaluated using a standardized questionnaire. Follow-up assessments 1 and 6 months after the intervention will examine long-lasting effects.

**Discussion:** The results of this study will provide insights into the changeability of social impairments in ASD by investigating social and emotional abilities on different modalities following repeated sessions of anodal tDCS with an intra-simulation training. Furthermore, this trial will elucidate the tolerability and the potential of tDCS as a new treatment approach for ASD in adolescents.

**Clinical Trial Registration:** The study is ongoing and has been registered in the German Registry of Clinical Trials (DRKS00017505) on 02/07/2019.

## Introduction

One out of 54 children fulfills the diagnostic criteria for autism spectrum disorder (ASD) (1), with boys being affected three to four times as often as girls (2). ASD is a neurodevelopmental disorder characterized by social and communicative deficits and repetitive, stereotyped behaviors, ranging over a wide spectrum with different degrees of impairment [International Classification of Diseases ICD-10; (3)].

As there is currently no cure for ASD, psychosocial and pharmacological treatments share the aim to ameliorate the core symptoms and enable a life as independent as possible (4). Psychosocial interventions show positive effects on intellectual and adaptive functioning (5), but suffer from limited support from randomized controlled trials (RCTs). Strongest evidence exists for the effectiveness of interventions that focus on early parent–child interactions (6, 7). Furthermore, there is no pharmacological treatment for the core symptoms of ASD and medication is mainly used for the therapy of commonly co-occurring disorders such as attention-deficit/hyperactivity disorder (ADHD) or depression (6). The need for efficient and affordable new treatment methods also becomes evident when assessing quality of life, employment, independency, social relationships, and mental and physical health in adults with ASD, which were found to be poor despite average cognitive functioning (8, 9). Thus, specifically targeting the mechanisms underlying the social–emotional dysfunctions in ASD can form the basis of new and viable treatment methods.

Social–emotional difficulties include pronounced impairments in empathy, a central prerequisite for social interactions. To be able to understand what others are feeling and to react appropriately to their emotions, it is often necessary to recognize the expressed emotion from others' faces. The ability to discriminate the six basic emotions (happy, sad, fear, anger, disgust, and surprise) is usually acquired in the first year of life (10), whereas the ability to discriminate more complex emotions improves until adulthood (11). It has been repeatedly shown that people with ASD have a deficit in the ability to recognize the emotions of others (12, 13). Moreover, results from neurological and electrophysiological studies show clear differences in automatic emotion recognition processes between ASD and typically developing children (14).

Another factor influencing social interactions is the direction of gaze (15). Eye-tracking reveals that children with ASD show aberrant patterns of gaze and fixation times when seeing human faces (16, 17) and eye gaze abnormalities have even been proposed as a robust biomarker for ASD (16). Moreover, dysfunctions in several brain regions including the dorsolateral prefrontal cortex (DLPFC) are involved in the development of this aberrant gaze behavior. In typically developing individuals, direct gaze leads to an enhanced response in brain areas important for empathic processes like theory of mind (ToM). In contrast, individuals with ASD show the same response when the gaze is averted and respond to direct gaze with an abnormal activation of the ToM network [medial prefrontal cortex (MPFC), temporoparietal junction, posterior superior temporal sulcus region, and amygdala] (15). These regions are also part of the social brain network (SBN) (18, 19).

Research regarding the neural underpinnings of behavioral social problems in ASD found evidence for alterations within the SBN in structural and functional magnetic resonance imaging (MRI) studies (20). Furthermore, structural MRI studies showed abnormalities in gray matter volume and white matter structure in short-distance tracts in the SBN, as well as an association between these abnormalities and social malfunctioning in ASD (21–24). Task-related fMRI studies in individuals with ASD revealed atypical activity and connectivity in social brain regions during the processing of social stimuli (25). For instance, processing of emotional facial expressions was associated with reduced activity in several brain regions important for interpersonal interactions, including the amygdala, MPFC, ventrolateral prefrontal cortex, and superior temporal sulcus region in individuals with ASD compared with typically developing individuals (26). Neuroimaging results show that the most consistently activated brain regions in ToM tasks are the medial prefrontal and orbitofrontal cortices (27). Within the ToM network, reduced functional connectivity in persons with ASD was detected (15). In detail, the functional connectivity between the MPFC and the posterior cingulate cortex (PCC) has repeatedly been shown to be lower in individuals with ASD (28, 29). A weaker connectivity between these regions is correlated with more serious social impairments [see (29)]. Both the MPFC and the PCC are part of the default mode network (DMN), which is involved in complex emotional and social processes such as ToM and self-referential thoughts (30, 31). Functional connectivity of DMN regions has been shown to be altered at rest and reduced in several social tasks such as face processing in adolescents and adults with ASD (28, 32). While the functional connectivity between the DMN nodes normally increases with age, the development of long-distance functional connectivity in the DMN cannot be observed in adolescents with ASD (28, 33). Also, brain regions important for social interactions outside the DMN exhibit reduced activity and functional connectivity during resting state (20). Individuals with ASD showed, compared with typically developing individuals, a weaker functional connectivity between the amygdala and the ventromedial prefrontal cortex (34) and a reduced connectivity within the lateral occipital cortex, which was associated with symptom severity (35). Thus, it appears that neurophysiologically based interventions should target the MPFC as a potential mediator for the reduction of ASD symptoms.

Contrary to fMRI, electroencephalography (EEG) allows for calculating the functional connectivity of the brain based on absolutely quantifiable rather than relative measures. In ASD, resting-state EEG studies suggest a reduced long-range connectivity between frontal lobe and other cortical regions and short-range overconnectivity (36, 37). Regarding frequency bands, decreased power in alpha is the most consistent finding (36, 38), which is thought to be associated with inhibitory control deficits in ASD (36).

Neuromodulation constitutes, alongside psychosocial interventions and psychopharmacology, the third pillar of therapeutic interventions in psychiatry. Transcranial direct current stimulation (tDCS) in particular is a non-invasive neurophysiological method, which has the potential to modify brain function by affecting neuronal resting membrane potentials (39, 40). It affects neuronal excitability on a sub-threshold level without eliciting action potentials (40–42). Depending on the goal of the intervention, anodal or cathodal stimulation can be used to either enhance or reduce neuronal excitability and thereby modulate processes related to the target brain region in the desired direction (43). The effects of direct current stimulation elicits a polarity-dependent facilitation or inhibition of the spontaneous neuronal firing rate (40, 43, 44). Post-stimulation effects of tDCS are contingent on the duration of the stimulation and last from several minutes up to some hours (40, 45). Studies investigating the effects of tDCS on neural activity showed that direct current can modulate cortical connectivity in the brain (46) and induce changes in the alpha frequency band during and after the stimulation (47–49). Moreover, fMRI studies found changes in brain connectivity in both task-related and resting-state networks after the application of tDCS (50–53). Brain stimulation is more effective and causes more long-lasting plastic changes in already activated neural circuits (54). Additionally, aftereffects of tDCS were shown to depend on the emotional and physiological state during stimulation (41). Given these findings, intra-stimulation engagement tasks are used to enhance the effects of tDCS [e.g., (55, 56)].

Therapeutically, tDCS is used to alter local neuronal excitability that is assumed to be in a dysfunctional hypo- or hyperactive state (45). Effects of the stimulation expand by changes in the neural network into more distant regions and lead to reorganization of neuronal circuits (45, 57). Due to encouraging results in the treatment of various neuropsychiatric disorders in adults (58–61), tDCS has gained attention in the treatment of childhood and adolescent neuropsychiatric disorders over the past years (45, 62–64).

Regarding the use of tDCS in children with ASD, 12 articles have been published to date (65–76). Among these, four are randomized, double-blind, sham-controlled studies; the remaining articles comprise experimental, quasi-experimental, and pilot studies, a case series, and a case study. Outcome measurements, number and details of stimulation sessions, and control conditions vary across the studies [for reviews see (77, 78)]. Therefore, the current evidence is sparse and methodologically incomparable (79). Nevertheless, these studies point toward positive effects of tDCS in the treatment of ASD showing an improvement in ASD symptoms such as socialization, sensory awareness, repetitive behaviors, and behavioral problems (65, 68, 70, 76) as well as in cognitive abilities often impaired in individuals with ASD (71, 74). The available results not only provide indications for significant symptom reductions maintained for 6 months (68) but also emphasize the necessity of further research (70, 79). The potential of tDCS to counteract ASD-specific aberrant brain activity was highlighted by studies showing modulation of long-distance connectivity (66), an increase in functional connectivity in the alpha band (68, 80), increasing connectivity between the hemispheres within alpha and induced neuroplastic changes (75). Moreover, studies report a reduction in ASD symptoms that were associated with an increase in the peak alpha frequency band in resting-state EEG (76) and increasing EEG complexity (72) after tDCS stimulation. These studies as well as the majority of the present tDCS studies in children and adolescents with ASD report reduced ASD symptoms after anodal stimulation with the anode placed over the DLPFC (77). However, none of the studies used a simulation-based approach to evaluate the peak magnitudes of the electric fields and to define the stimulation target. TDCS applied over this commonly used stimulation site is able to increase the functional connectivity between the MPFC and the PCC in healthy adults (51) as well as in individuals with ASD (81). Taken together, both theoretical considerations and empirical findings regarding the pathophysiological signature of ASD lead to the proposition that tDCS can be used to alter dysfunctional patterns of brain activity.

The goal of the present study is to investigate the clinical and scientific significance of tDCS for improving social cognition abilities in ASD with a clear neurobiological rationale. Therefore, the planned study will (1) examine tDCS-related changes on reported symptomatic changes, behavior, brain activity, and connectivity; and (2) explore the long-term effects of tDCS in ASD and thereby evaluate tDCS as a cost-effective, time-efficient treatment option for children and adolescents with ASD.

## Methods and Analysis

The study is designed as a randomized, double-blind, sham-controlled clinical trial with two follow-up measurements 1 and 6 months after the last stimulation session, respectively.

### Participants

Measurements and interventions will be carried out at the Department of Child and Adolescent Psychiatry at the Medical University of Vienna, Austria. The participants will be recruited from the outpatient clinic and from local institutes for individuals with ASD.

#### Sample Size Calculations

Power analysis was done with G^*^Power version 3.1.9.2 (82) to detect a difference in the primary outcome [SRS, (83)] calculating repeated measures ANOVA considering an effect size *f* of 0.25, a power of 80%, and an α of 0.05. Previous studies in children with ASD have shown larger effect sizes for tDCS (63). A sample size of 20 participants (*N* = 20) will be necessary to report medium effect sizes on the primary outcome. Analyses of secondary outcomes were not included in the power analysis and therefore considered underpowered.

#### Randomization

A total of 20 male participants will be included. To balance the experimental and control group with respect to comorbidities, all participants will be stratified into one of three subgroups based on their clinical presentation: subgroup A for participants without comorbidity, subgroup B for participants with depression as primary comorbidity, and subgroup C for participants with ADHD and/or conduct disorder as primary comorbidity. After subgroup stratification, the participants will be randomly allocated to the active condition or the sham condition by assigning a code to each participant. To ensure a quick study start for the participants after enrollment, a block randomization design will be used.

#### Blinding

The study mode of the stimulation device encodes sham and active stimulation using preprogramed codes so that the participant and investigators will be blind to the type of stimulation. The stimulation parameters (duration, fade-in, fade-out time, and current intensity) are set and saved on the device to avoid accidental modification of the parameters. To investigate the success of the subject-blinding, participants will be asked about their beliefs of the received stimulation type at the last stimulation session. After completing the 6-month follow-up measurement or after dropping out of the study, participants, and their caregiver will be informed about group allocation. Participants in the sham condition will not receive active stimulation after the end of the trial. Premature unblinding using prepared envelopes can be performed when knowledge of the actual group allocation becomes necessary for the safety of the patient. The blinding, code storage, and preparation of code-break envelopes will be done by a staff member not directly involved in the intervention and the day-to-day management of the study.

#### Inclusion Criteria

Participants eligible for the trial must comply with all of the following:

- Male
- Fulfilling ICD-10 criteria for ASD and diagnosed with ASD from a trained professional using the Autism Diagnostic Interview-Revised [ADI-R; (84)] and/or the Autism Diagnostic Observation Schedule [ADOS 2; (85)]
- Age between 12 and 18 years
- Right-handed
- IQ ≥ 70 assed using standardized instruments
- Treatment-naive to neurostimulation
- Signed written informed consent of the participant and primary caregiver.

#### Exclusion Criteria

- Contraindications for tDCS such as cardiac pacemakers, defibrillator, cochlear implant, intracranial/cranial stimulators, and other metals in the head
- Contraindications for MRI
- Epilepsy or related seizure disorders
- Other severe neurologic or psychiatric disorders or medical conditions (e.g., skull defect and craniotomy)
- Concomitant psychopharmacological medication.

Ongoing concomitant social or psychotherapeutic long-term interventions must be held constant during the course of the intervention.

### Intervention

**Figure 1:**
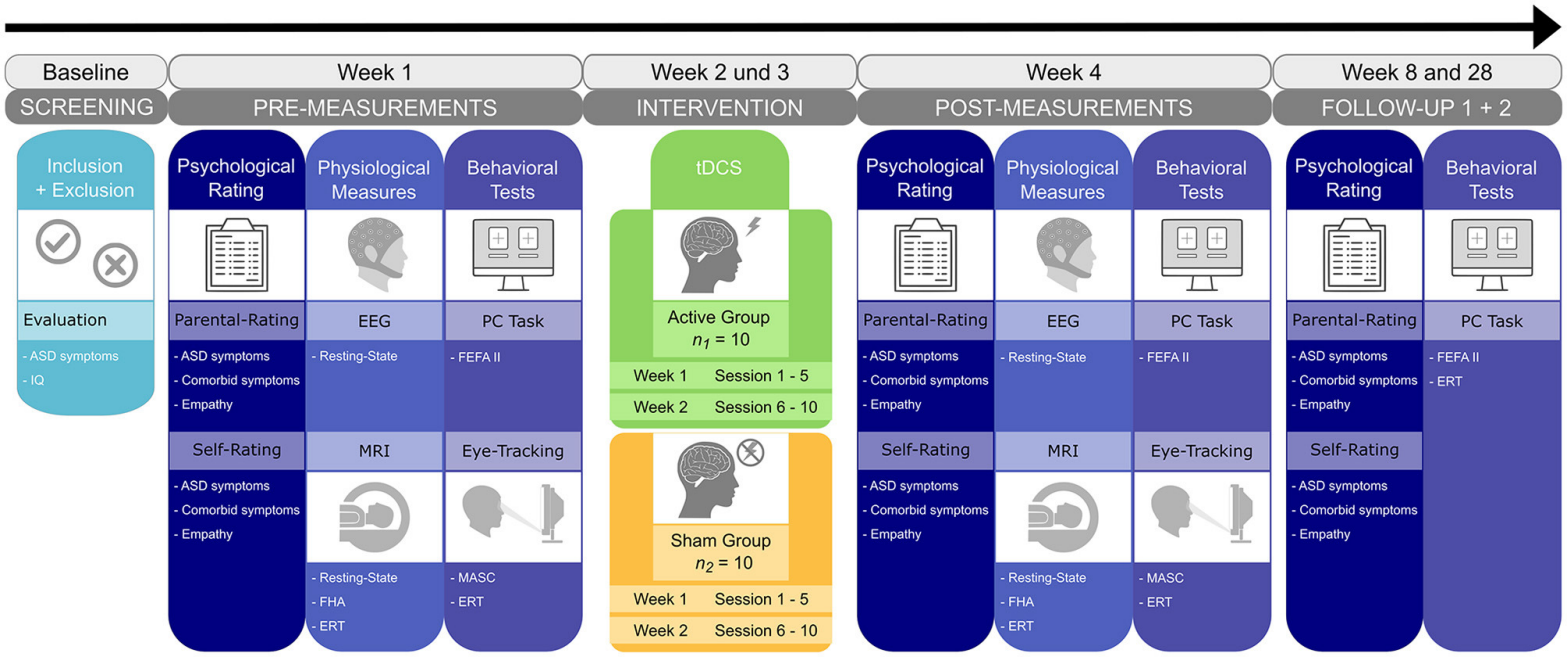
Study timeline and overview. After enrollment, participants will undergo a set of psychological, behavioral, and physiological baseline measures in the first week. Following randomization, 10 sessions of either active or sham tDCS will be conducted within a 2-week interval. Post-treatment measures are repeated on the subsequent week. Two follow-up measures are planned 1 and 6 months after the end of the tDCS intervention. ERT, emotion recognition task; FEFA II, frankfurter test and training for recognizing facial affect; FHA, frith-happé animations; MASC, movie for the assessment of social cognition.

The intervention phase comprises 10 sessions of tDCS or sham stimulation within two consecutive weeks, with a 2-day break after the fifth session (see Figure 1 for study overview). If feasible for the participant, all stimulation sessions will be held at the same time of the day. In each session, prior to the stimulation, the participant will complete a brief questionnaire about general well-being, attention, motivation, and experiences before the session. Afterwards, two rubber electrodes with conductive paste will be attached to the head of the participant. To decide on the optimal stimulation parameters, an MRI-based finite-element model approach was utilized, which simulates the intensity, distribution, and focality of the electric field induced by tDCS. To this aim, SimNIBS 3.1 (86) and structural T1 images of adolescents with ASD from a previous clinical trial executed by our research group were used. The parameters under comparison comprised an intensity of 2 mA, three different electrode sizes (3 × 3, 5 × 5, and 5 × 7 cm rectangular rubber electrodes) and two different conductors (NaCl solution and Ten20 paste).

**Figure 2:**
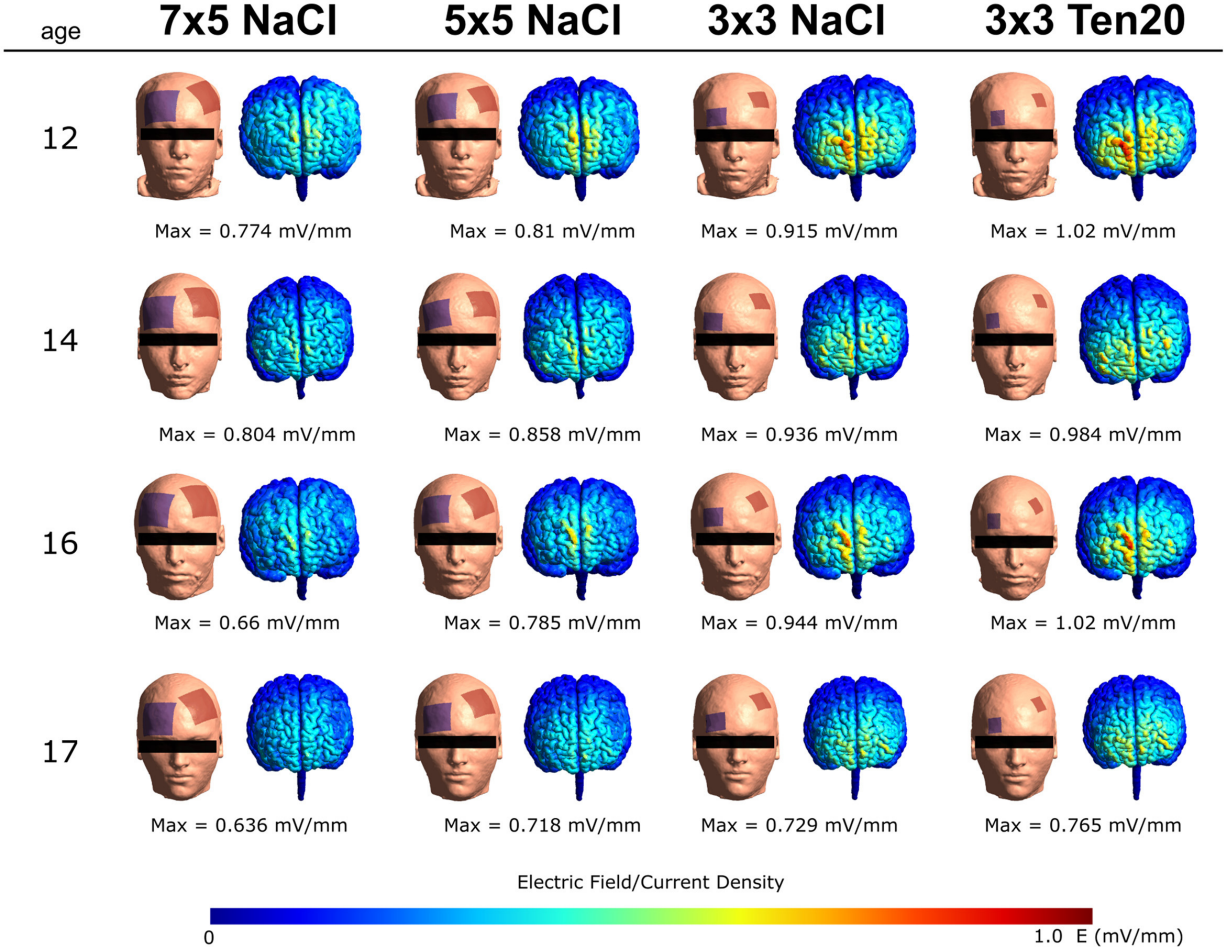
Four examples of the simulations of our tDCS protocol in adolescents using SimNIBS and previously acquired T1-weighted MRI scans of a comparable population. The electric field was simulated using bilateral montages (anode at F3 and cathode Fp2-supraorbital), an intensity of 2 mA, and three different electrode sizes. Each row represents one participant and each column represents one montage. Peak magnitudes of the intracranially generated electric fields are listed below each simulated configuration.

Simulations demonstrated peak intensity of electric fields showing stronger fields with smaller electrodes (3 × 3 cm) and conductive Ten20 paste. Here, stimulating anodal tDCS over the left DLPFC would lead to peak magnitudes at the MPFC (see Figure 2), which match with the targeted neural circuit. This stimulation setup is in line with the recommendations suggested in a recent systematic review and meta-analysis on the existing literature of tDCS in ASD (77) and with other studies demonstrating effects of tDCS on reducing ASD symptoms. Therefore, an electrode montage with the anode over the left DLPFC (F3 according to the international 10-20 system for EEG) and the cathode over the right supraorbital region will be used in the current trial.

To localize the electrode site F3, the Beam F3 system (87) will be used. Anodal tDCS will be administered through an Eldith-DC Stimulator (NeuroConn GmbH, Germany) at 2 mA current strength for 20 min with a fade-in and fade-out of 30 s at the start and end of the stimulation. During the sham stimulation, a fade-in phase of 30 s, followed by 40 s of 2 mA stimulation and a fade-out phase of 30 s will be applied. The participants will experience the typical skin sensation usually produced at the beginning of an active tDCS stimulation and are therefore expected to remain unaware of the real condition (88).

#### Safety and Adverse Events

As for potential risks of tDCS, guidelines report no serious adverse events in over 18,000 sessions of low-intensity transcranial electrical stimulation in pediatric and adult patients, as well as in healthy subjects (89). Current data also specifically emphasize the feasibility, tolerability, and safety of tDCS in children and adolescents (45, 63, 90). Adverse effects of tDCS in children and adolescents were described as mild and transient, including tingling (11.5%), itching (5.8%), redness (4.7%), and scalp discomfort (3.1%) (91). Thus, when guidelines are followed, tDCS is a safe modality for children and adolescents with various neurological conditions (92). The proposed parameters of this study (including electrode size, current strength, and duration) were previously tested for their safety in children younger than the participants in this study (93, 94). The brain stimulation device will be operated by experienced psychologists, neuroscientists, and research assistants trained in the application. Therefore, no serious adverse events or serious health risks for the participants of this study are expected. In the case of a serious adverse event, the participant will be excluded from the trial and the adverse event will be reported to the ethics committee of the Medical University of Vienna and to the Austrian Agency for Health and Food Safety (AGES). To protocol adverse effects, the German version of the questionnaire of sensations related to transcranial electrical stimulation will be used at the last stimulation session (accessible at: http://neurologie.uni-goettingen.de/downloads.html).

### Instruments

In accordance with the complexity of the disorder, the effects of the tDCS intervention will be investigated at three different levels:

1. Psychological ratings to detect changes in ASD symptoms as well as in comorbid symptoms;
2. Neurophysiological measures to detect changes in brain responses to emotional stimuli;
3. Behavioral tests to detect changes in responses and gaze behavior.

#### Psychological Ratings

Clinical information *via* questionnaires will be collected from participants and caregivers following a multi-perspective approach. The Social Responsiveness Scale [SRS; (83)] reliably indicates the presence and severity of social impairment in ASD and correlates with real-world dysfunctional behaviors. Five subscales (social awareness, social cognition, social communication, social motivation, restricted interests and repetitive behavior) and a total score will be calculated. Additionally, various instruments targeting different aspects of ASD symptoms [Autism Treatment Evaluation Checklist—ATEC (95); Social Communication Questionnaire—SCQ (96)] will be used for pre-, post-, and follow-up assessments. Furthermore, affective and cognitive components of empathy and emotion regulation abilities will be measured *via* self-reports [Emotion Regulation Questionnaire—ERQ (97); Index of Empathy for Children and Adolescents—IECA (98); Basic Empathy Scale—BES (99)] and parental questionnaires [Griffith Empathy Measure—GEM (100); Emotion Regulation Checklist—ERC (101)]. For subgroup stratification and the investigation of comorbid symptoms of ASD such as attention deficit, anxiety, aggression, and depression, the Diagnostic System for Mental Disorders in Childhood and Adolescence for ADHD, Depression, Anxiety, and Conduct Disorder [DISYPS III ADHS, DEP, ANG, SSV—self- and parental rating versions; (102)] will be used.

#### Behavioral Tests

**Frankfurter test and training for recognizing facial affect (FEFA II):** This computer-based program was developed to train and reliably test the recognition of facial affect in individuals with high-functioning ASD (103). Participants are asked to identify the six basic emotions and neutral expressions presented in 50 black-and-white photographs of faces. The stimuli used are based on Ekman's conceptualization of basic emotions (104). Correct classification and reaction times are measured.

**Movie for the assessment of social cognition (MASC):** The German version of the MASC (105) is a reliable and sensitive naturalistic measure of social cognition and gaze behavior in adolescents with ASD (106). The MASC consists of a film showing two females and two males having a dinner party. The film lasts about 15 min and is paused 43 times to ask questions about the actors' emotions, thoughts, and intentions. The revised MASC score is a measure of the degree of social cognition adopted for adolescents and will be calculated from the correct responses (106). Additionally, eye-tracking data of the participants' gaze behavior will be recorded during the presentation of the movie using the Tobii Pro eye-tracker (Tobii Group, Danderyd, Schweden) and Tobii Studio 3.4.5 (107).

**Figure 3:**
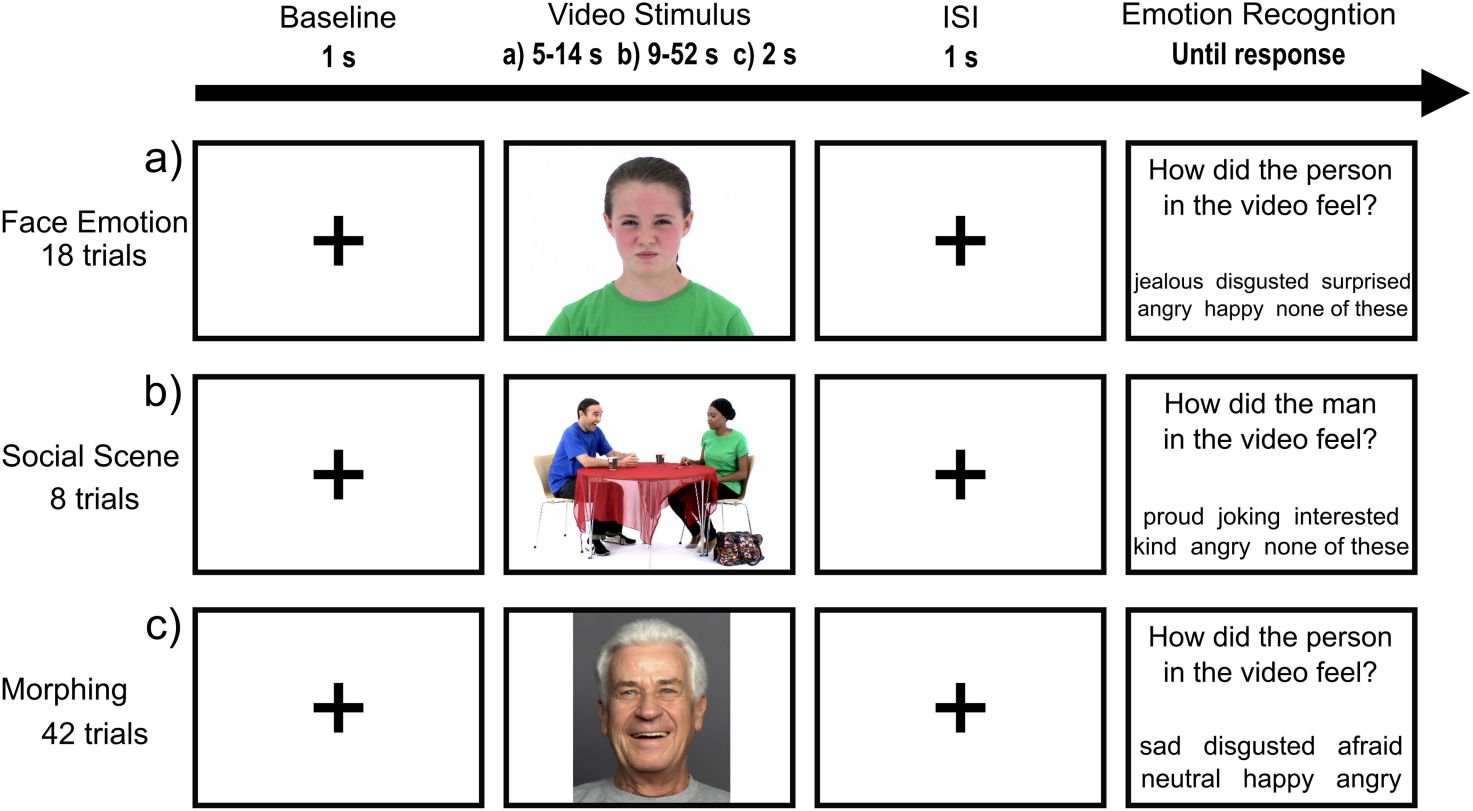
Structure of ERT for all three parts. After the baseline (crosshair), the emotional stimuli will be presented followed by an interstimulus interval and the emotion recognition question. Part **(a)** shows videos of one person expressing a basic or complex emotion; **(b)** presents two or more people engaging in an emotional social scene; **(c)** consists of morphing videos.

**Emotion recognition task (ERT):** This self-developed task uses videos of faces and social scenes depicting different emotional states to investigate emotion recognition skills. The task involves three parts (see Figure 3 for a graphical illustration) addressing different emotion recognition competencies: (a) shows videos of one person expressing a basic or complex emotion; (b) is more challenging and presents a more naturalistic scenario in which two or more people engage in an emotional and dynamic social scene; (c) consists of morphing videos (displaying a face transitioning from a neutral expression to a basic emotion) and participants will be instructed to interrupt the morphing process as soon as they recognize the displayed emotion. Afterwards, participants will be asked to classify the emotion that was displayed in the video. The emotional stimuli represent all age groups and genders equally and are taken from validated databases. Parts (a) and (b) use stimuli form the EU Emotion Stimuli Set (108, 109), while part (c) uses stimuli from the FACES Database (110, 111). This task was implemented in Python (version 3.6.7) using the PyQt (version 5.15.2) graphics library. The ERT will be employed in pre- and post-measurements to examine changes in correct classifications and response times and to investigate pre–post changes in gaze behavior using the Tobii Pro eye-tracker (Tobii Group, Danderyd, Schweden).

**Pre-stimulation task:** Prior to each stimulation session, the participants will be measured on accuracy of performance and response time on the ERT. This pre-stimulation task without tDCS functions as a reference to compare with the intra-stimulation task, which is presented during tDCS. This comparison will provide information about the acute effects of tDCS on emotion recognition abilities.

**Intra-stimulation training:** During the 20 min of active or sham tDCS, all participants will undergo a structured training that aims to further stimulate the engagement of the targeted circuits during the active and sham tDCS sessions. At the first 5 min of the stimulation, the child-friendly movie *Inside Out* (112), which focuses on emotions and was developed with scientific consultants studying emotions, will be presented. The movie will be interrupted various times to ask questions about the mental state and emotions of the characters. Next, the ERT will be completed by the participants to further stimulate the engagement of the targeted circuits. Afterwards, the next part of the movie, again interrupted with questions, will be presented. This approach will ensure that all participants will be dealing with the same material and therefore are assumed to engage similar brain regions during the stimulation. Furthermore, this repeated examination of emotion recognition performance with the ERT will provide important information about learning curves and session-by-session changes, which may reveal insights into the optimal number of stimulation sessions.

#### Neurophysiological Measures

**EEG data acquisition:** EEG data will be collected from 19 cortical sites (Fp1, Fp2, F3, F4, F7, F8, Fz, FCz, C3, C4, Cz, T5, T6, P3, P4, Pz, O1, O2, and Oz) positioned according to the international 10-20 system using Ag/AgCl electrodes and the Thera Prax Q-EEG System (NeuroConn, Illmenau, Germany). Electrode-skin impedance will be kept below 3 kΩ.

**Resting-state EEG:** Neural oscillations will be recorded in eight 1-min trials, four with eyes-closed (C) and four with eyes-open (O). A guideline-based counterbalanced order (COCO-pause-OCOC) will be used considering disorder-specific characteristics such as a discomfort with longer eyes-closed intervals and minimizing the risk of participants falling asleep in the eyes-closed condition (113, 114).

**MRI data acquisition:** Functional and structural whole-brain imaging will be conducted using a 3-T Siemens MAGNETOM Prisma MR Scanner (Siemens, Erlangen, Germany) equipped with a 64-channel head coil.

**Resting-state fMRI:** RS data will be acquired for 7 min using an echo-planar imaging sequence with the following parameters: TE/TR = 35/728 ms, field of view (FoV) = 192 × 192 × 144 mm, matrix size = 96 × 96, 72 slices, multiband factor = 8, resulting in an isotropic voxel size of 2 mm, flip angle = 55° (optimized Ernst angle), and bandwidth = 3,365 Hz/Px [sequence optimized according to (115)]. To reduce head motion and participant drowsiness, the movie *Inscapes* (116) will be shown during resting-state recordings. This non-social movie features abstract shapes and was developed for the use with children and clinical populations to increase compliance while minimizing cognitive demand (116).

**ToM-task: frith-happé animations (FHA):** To investigate changes in the ability to infer people's thoughts and feelings, a validated ToM-task, the FHA, will be used (117–119). Participants will be instructed to categorize 12 video clips depicting two animated triangles as showing either no interaction/random movements, physical interaction/goal-directed behavior, or emotional interaction/ToM after each video. Additionally, after the four ToM trials only, they need to state how each of the triangles felt at the end of the video using visual analog scales (VAS). VAS are used instead of the original good/neutral/bad decision to allow for more fine-grained inferences. For each decision (i.e., the video category and emotional state of each triangle), subjects are given 5 s, before the paradigm continues, even when no answer was given. One trial contains up to three baseline (BL) periods: (1) Before the videos, BLs are uniformly jittered between 7 and 13 s. (2) Before the category choice, the BL is between 2 and 4 s. (3) In case a ToM video was shown, the BL before the VAS is 6 s minus the duration for 2.

**Emotion recognition task:** Parts (a) and (c) of the ERT (see Figure 3) will be presented during MRI using a jittered and randomized baseline duration between (a) 9–15 s and (c) 5–10 s. Imaging is conducted using the same parameters as for the resting-state recordings.

**Structural MRI:** These measures will be performed to exclude previously unknown alterations in brain anatomy using the following parameters: T1: TE/TR/TI = 2.29/2,300/900 ms, FoV = 165.44 × 240 × 240 mm, matrix size = 256 × 256, 176 slices, 0.94 × 0.94 × 0.94 mm voxel size (rounded), flip angle = 8°, bandwidth = 200 Hz/Px; T2: TE/TR = 408/3,200 ms, FoV = 172.8 × 230 × 230 mm, matrix size = 256 × 256, 192 slices, 0.9 × 0.9 × 0.9 mm voxel size (rounded), flip angle = 120°, bandwidth = 725 Hz/Px. During the structural scans, cartoons will be shown to the participants.

### Outcomes

The primary outcome will be a reduction in ASD symptoms from baseline in the experimental group compared to the sham group. ASD symptoms will be quantified by the raw score of the German version of the SRS (83) assessed by the primary caregiver.

The secondary outcomes will be increased social cognition and emotion recognition quantified by the raw scores of the MASC (105) as well as correct classifications in the ERT and the FEFA 2 (120). Regarding gaze behavior, longer fixation duration on the eyes and increased pupil dilation will be the outcomes on the MASC and ERT. Outcomes on participants and caregiver ratings will be a decrease in ASD symptoms measured with the ATEC (95) and SCQ (96) and an increase in empathy and emotion regulation abilities measured with the ERQ (97), IECA (98), BES (99), GEM (100), and ERC (101). Neurophysiological activity will be measured at baseline and after the intervention using resting-state EEG and fMRI to measure changes in functional connectivity and electrical activity in the brain. We expect an increased resting-state alpha-power, a decrease in the other frequency bands, and an increased connectivity within the ToM network and DMN. An increased regional activation in key areas of the SBN will be the outcomes during the FHA and ERT.

The exploratory outcomes will include participant and caregiver ratings on comorbid symptoms of ASD using the DISYPS III (102). The brain activation in regions involved in emotion recognition processes and cognitive empathy during emotion recognition with the ERT during fMRI will also be examined exploratorily.

### Statistical Analysis

For the statistical analyses, repeated measures analysis of variance (ANOVA) and hierarchical linear models (HLMs) will be used. A 4 × 2 mixed ANOVA (primary outcome as dependent variable) with *Time* (pre vs. post vs. follow-up 1 vs. follow-up 2) as the within-subjects factor and *Group* (experimental vs. control group) as the between-subject factor will be conducted to investigate an interaction between time and group. In order to test specific effects, *post-hoc* tests will be employed. A HLM will be formulated to investigate time-dependent changes and group differences in task performance across stimulation sessions. A *p* < 0.05 will be considered statistically significant. Analysis will be conducted in *R* (121).

For the eye-tracking data, analysis of fixation duration and pupil dilation will be done in Tobii Studio 3.4.5 (107) following the preprocessing pipeline of other studies using eye-tracking to investigate emotion recognition and social cognition in adolescents with ASD [for the ERT (17) and for the MASC (106)]. These results in a fixation duration on the specified area of interest (AOI) and a measure of average pupil dilation per video. Further analysis regarding pre–post comparisons of AOIs and pupil dilation will be done in *R* (121) using linear models.

For the EEG data, we will employ the *MNE* library (122) written in *Python* (123) to execute data pre-processing, segmentation, and frequency and connectivity analyses. The raw EEG data of each participant will be segmented into epochs of 2 s. A multi-taper method with adaptive weights will be applied to each epoch to obtain power spectral density (PSD) values in each frequency band of interest. Frequency bands of interest will be delta (0.5–4 Hz), theta (4–8 Hz), and alpha (8–12 Hz). Subsequently, PSD values in each frequency band will be averaged across epochs separately for each participant, measurement occasion, and experimental condition.

Basic fMRI processing will be conducted according to (124) using Statistical Parametric Mapping [SPM; (125)]. To counteract the expected high level of in-scanner motion, PESTICA/SLOMOCO (126, 127) and the BrainWavelet toolbox (128) will be used for additional movement and artifact correction. Spatial normalization will be performed to a custom template created using the CerebroMatic toolbox (129) to avoid implausible deformation to match the usual standard adult brain. Subject-level analysis will be conducted using the general linear model in SPM. The FHA task will be modeled according to (118, 130). The ERTs will be modeled as block designs with a single condition. For the morphing variant, the reaction times will be included as a parametric modulator to uncover the possible influence of the subjective strength of the perceived emotion. The RS data will be analyzed by means of seed-based functional connectivity. Since the ERTs constitute newly implemented paradigms, interesting regions of their baseline activation will be used as seeds here. Nuisance regressors for the linear models will be derived *via* the CompCor (131) and Friston-24 (132) approaches with an additional bandpass applied to the RS data. Group-level analyses of fMRI data will be conducted using the Flexible Factorial module in SPM. The time-by-treatment interaction effects will be tested at *p* < 0.05 family-wise error corrected at the peak level and with a primary threshold of *p* < 0.001 uncorrected at the cluster level.

## Discussion

This study protocol presents the design of an RCT investigating the therapeutic effects of repeated sessions of anodal tDCS over the left DLPFC using a combined approach of EEG, fMRI, and eye-tracking data as well as clinically relevant scores from participants and caregivers. Therefore, the results of this study will elucidate the changeability of aberrant activity through neurostimulation on multiple levels.

An important determinant of stimulation effectiveness is the placement and size of the electrodes (133, 134). Accordingly, and in contrast to previous tDCS studies in children and adolescents with ASD, our study utilizes a parameter configuration based on computational modeling using MRI scans from adolescents with ASD for the simulations.

Another contrast to previous investigations is the use of an intra-stimulation training aiming specifically at improving social cognition and emotion recognition. The training is designed to not only motivate the participants and standardize what they are doing during the stimulation, but also to evoke activity in the target regions of the stimulation. To account for different levels of skills among the participants and to increase ecological validity, the task involves emotion recognition at different levels of difficulty using basic and complex emotions that will be presented isolated or in a dynamic social scene. Moreover, the repeated examination of emotion recognition performance will provide important information about learning curves and session-by-session changes. This may guide future projects in deciding about the ideal number of stimulation sessions, as studies investigating the cumulative effects of tDCS over several days are sparse. Furthermore, there is not much evidence available about the long-term effects of this treatment method. By including two follow-up measures, this study will provide information about the persistence of treatment effects regarding the clinical symptomatology up to 6 months after the end of the intervention.

Within this project, it will also be monitored how the participants react to the stimulation using the questionnaire of sensations related to transcranial electrical stimulation. Even though the safety of tDCS in pediatric populations has been demonstrated repeatedly (92), information about how this patient group perceives and tolerates the mild adverse events of tDCS seems warranted. Individuals with ASD often suffer from sensory hypersensitivity (135) and may thus respond differently to the stimulation.

In this trial, a standard bipolar tDCS montage will be used with the anode placed over the DLPFC targeting the MPFC. However, other montages or stimulation methods such as multi-electrode tDCS or repetitive transcranial magnetic stimulation (rTMS) are also of interest for this population. Here, this established bipolar direct current protocol was chosen as it has milder side effects, has no history of reported serious adverse effects, and is noiseless compared to rTMS (91, 92, 136, 137). Therefore, it might be better tolerated in individuals with ASD, and participants will not be distracted from the intra-stimulation training by potentially painful sensations or sound. Regarding the usage of tDCS as a clinical treatment, a bipolar tDCS has the advantage of being more affordable, portable, and easy to administer, and it opens the possibility for home use in the future. A comparison of the effects of different stimulation protocols as well as stimulation sites is an essential element for future studies.

During the intervention, participants will have daily contact with the study team and undergo the intra-stimulation training. Although these factors could have a beneficial effect on ASD symptoms and emotion recognition skills, the double-blind design and randomization to either active or sham tDCS treatment should mitigate this potential bias.

In conclusion, children and adolescents with ASD are in need of an effective treatment, and modulating brain activity could be a first step toward a new efficient intervention. This neurophysiologically based approach benefits from being non-invasive, being easy to administer, and having only mild and transient side effects (92, 138). TDCS further aims to overcome shortcomings of traditional interventions, such as often long and expensive treatment periods (5). Taken together, this study will provide information about the efficacy of tDCS and the potential to establish it as an affordable alternative or additive treatment for individuals with ASD in the long run.

## Trial Status

The described trial is ongoing, and recruitment commenced in July 2019. Recruitment and interventions had to be paused due to COVID-19 restrictions from March 13, 2020 to June 15, 2020. The data collection will continue until 20 participants completed all 10 intervention sessions and the corresponding pre- and post-measurements. Follow-ups will be conducted 1 and 6 months after the stimulation sessions to investigative long-lasting effects.

## Ethics Statement

The study was approved by the ethics committee of the Medical University of Vienna and the Austrian Agency for Health and Food Safety (AGES) and will be conducted according to the Declaration of Helsinki. Both participants and caregivers receive information sheets and consent forms about the study to take home. Participants and caregivers must agree to take part in the study and return their signed informed consent forms, before being included in the study. Consent can be withdrawn by the participants or caregivers at any time without any effect on their standard clinical treatment.

## Author Contributions

KP, LK, LP, and PP designed the trial. KP and SR developed, programmed, and piloted the ERT paradigm. GA, KP, and LK designed the stimulation model and defined the stimulation parameters. MK, RL, SR, and KP planned the data analysis. KP and LK coordinate the recruitment of the participants. KP and MK are responsible for the data collection during the study. KP, SR, GA, MK, RL, PP, LP, and LK wrote, edited, and revised the paper. All authors approved the final manuscript.

## Conflict of Interest

RL received travel grants and/or conference speaker honoraria within the last 3 years from Bruker BioSpin MR and Heel and has served as a consultant for Ono Pharmaceutical. He received investigator-initiated research funding from Siemens Healthcare regarding clinical research using PET/MR. He is a shareholder of the start-up company BM Health GmbH since 2019. The remaining authors declare that the research was conducted in the absence of any commercial or financial relationships that could be construed as a potential conflict of interest.

## Publisher's Note

All claims expressed in this article are solely those of the authors and do not necessarily represent those of their affiliated organizations, or those of the publisher, the editors and the reviewers. Any product that may be evaluated in this article, or claim that may be made by its manufacturer, is not guaranteed or endorsed by the publisher.
